# *Cryptosporidium felis* and *C. meleagridis* in Persons with HIV, Portugal

**DOI:** 10.3201/eid1012.031068

**Published:** 2004-12

**Authors:** Olga Matos, Margarida Alves, Lihua Xiao, Vitaliano Cama, Francisco Antunes

**Affiliations:** * Instituto de Higiene e Medicina Tropical, Lisboa, Portugal;; † Centers for Disease Control and Prevention, Atlanta, Georgia, USA;; ‡ Hospital de Santa Maria, Lisboa, Portugal

**Keywords:** letter, Cryptosporidiosis, unusual Cryptosporidium species, HIV-infected patients, Portugal

**To the Editor:** *Cryptosporidium*, a pathogenic protozoan parasite with a worldwide distribution, causes diarrheal illness in humans and animals. The parasite can be transmitted from human to human through fecal-oral contact (household contact and nosocomial transmission), sexual contact, ingestion of contaminated food or water, and contact with infected animals. Molecular diagnostic methods indicate that *Cryptosporidium parvum* and *C. hominis* are the major causes of cryptosporidiosis in humans, and other *Cryptosporidium* species can be associated with human infection (*1**–**5*).

In Portugal, patients with AIDS have an 8% prevalence rate of cryptosporidiosis (*6*) with *C. parvum* and *C. hominis* as the etiologic agents, even though other *Cryptosporidium* species were found in these patients (*3**,**7**,**8*). This study characterizes clinical manifestations of infections with unusual two different *Cryptosporidium* species isolated from seven patients and demonstrates that these species can cause life-threatening disease.

Cryptosporidiosis was diagnosed in 40 patients from 1994 through 2002. All patients were serologically positive for HIV-1 and had diarrhea (at least two loose stools per day) when diagnosed with cryptosporidiosis. Demographic, clinical, and immunologic data were obtained from each patient's records. *Cryptosporidium* oocysts were identified by light microscopy after concentration from fecal material by a modified water-ether sedimentation method followed by the modified Ziehl-Neelsen staining (*7*). The intensity of infection was quantified before molecular analysis by scoring the number of oocysts counted per microscopic field (under a 20x objective) of 50-μL volume of concentrated stool sample as + (1–5 oocysts), ++ (6–10 oocysts), +++ (11–15 oocysts), or ++++ (>15 oocysts). Genetic characterization of the isolates was based on polymerase chain reaction–restriction fragment length polymorphism analysis of the small subunit rRNA gene (*2**,**3*).

The molecular analysis showed that 22 patients (55%) were infected with *C. parvum*, 11 (27.5%) were infected with *C. hominis*, 4 (10%) were infected with *C. felis*, and 3 (7.5%) were infected with *C. meleagridis*. Of the four patients infected with *C. felis*, three (75%) showed low (+) and one (25%) showed moderate (++) oocyst loads. All three (100%) patients infected with *C. meleagridis* showed low oocyst loads (+). In contrast, of the 22 patients infected with *C. parvum*, 9 (41%) showed low oocyst loads (+), 3 (14%) showed moderate oocyst loads (++), 3 (14%) showed high oocyst loads (+++), and 7 (32%) showed very high (++++) oocyst loads. Similarly, of the 11 patients infected with *C. hominis*, 2 (18%) had low oocyst loads (+), and 3 (27%) each had moderate (++), high (+++), or very high (++++) oocyst loads.

Five of the seven patients infected with *C. felis* and *C. meleagridis* were men and two were women; the median age of patients was 31 years (7–44 years). In this group of HIV-positive patients, three were heterosexual persons, two were homosexual persons, one was an intravenous drug user, and one acquired HIV infection through vertical transmission. Of the seven patients, all showed a range of clinical manifestations of infection, including transient diarrhea, chronic diarrhea, dehydration, and cachexia. Five (71%) of the patients spontaneously recovered, and two (29%) of the patients died. The median CD4^+^ count/mm^3^ was 20 (range 18–213).

All of the seven patients were prescribed antiretroviral therapy, but one of the patients did not adhere to the treatment. Two of the three patients infected with *C. meleagridis* died of cryptosporidiosis. Information on the risk factors for acquiring *Cryptosporidium* infection was available for one patient, the child infected with *C. felis*, who had contact with cats at home. No other potential intestinal pathogens were detected in the feces of these patients at the time of the cryptosporidiosis diagnosis.

Twenty-two of the 33 patients infected with *C. parvum* and *C. hominis* were men and 11 were women; the median age of patients was 32 years (7–58 years). Sixteen patients were intravenous drug users, 5 patients were heterosexual persons, 1 patient was a homosexual person, and 2 patients acquired HIV infection through vertical transmission; the remaining 9 patients had no HIV-exposure history information. Eighteen of the 22 patients infected with *C. parvum* showed a range of clinical manifestations of illness with transient diarrhea, chronic diarrhea, dehydration, and cachexia. Twelve (67%) of the patients spontaneously recovered, and 6 (33%) of the patients died. Information on CD4^+^ count/mm^3^ was available for 13 of the 22 patients with a median count of 20 (range 3–250). Information on the outcome of the patients infected with *C. hominis* was available for 10 of the 11 patients. All of the 10 patients showed a range of clinical manifestations of infection, including transient diarrhea, chronic diarrhea, dehydration, and cachexia. Seven (70%) of the patients spontaneously recovered, and 3 (30%) of the patients died. The median CD4^+^ count/mm^3^ was 20 (range 6–40).

Most reports on infections with unusual *Cryptosporidium* species in humans give a brief description of the genotyping results, leaving the clinical importance of these species uncertain. Unusual *Cryptosporidium* species can cause disease (symptomatic infection) and death. *C. felis* and *C. meleagridis* infections showed low oocyst shedding (all seven patients had low to moderate oocyst loads in samples). On the contrary, *C. parvum* produced similar clinical manifestations but showed higher oocyst shedding; 46% had high to very high parasite loads. *C. hominis* infections had parasite loads even higher than *C. parvum* infections; 54% of patients had high to very high parasite loads. In immunocompetent persons, *C. hominis* infections produce higher oocyst loads in feces than infections caused by *C. parvum* or zoonotic species (*2**,**9*).

The transmission route for the unusual *Cryptosporidium* species is unclear. Because human infection by unusual *Cryptosporidium* species is less common, the principal transmission route for these parasites is likely through direct contact with infected animals. In our study, one of the four immunocompromised patients with *C. felis* was a child who had been in close contact with cats at home. No data on animal contact were available for other patients infected with unusual *Cryptosporidium* species. Cats are found in many homes with no evidence of cryptosporidiosis; therefore, it is difficult to attribute the occasional human *C. felis* infection to contamination by cats. Careful epidemiologic studies are needed to elucidate the transmission route of human infections with unusual *Cryptosporidium* species.
